# RFX3 Modulation of FOXJ1 regulation of cilia genes in the human airway epithelium

**DOI:** 10.1186/1465-9921-14-70

**Published:** 2013-07-03

**Authors:** Lukas Didon, Rachel K Zwick, Ion Wa Chao, Matthew S Walters, Rui Wang, Neil R Hackett, Ronald G Crystal

**Affiliations:** 1 Department of Genetic Medicine, Weill Cornell Medical College, 1300 York Avenue, Box 164, New York New York 10065, USA

**Keywords:** Lung epithelium, Ciliated cell differentiation, Human, FOXJ1, RFX3, Basal cell

## Abstract

**Background:**

Ciliated cells play a central role in cleansing the airways of inhaled contaminants. They are derived from basal cells that include the airway stem/progenitor cells. In animal models, the transcription factor FOXJ1 has been shown to induce differentiation to the ciliated cell lineage, and the RFX transcription factor-family has been shown to be necessary for, but not sufficient to induce, correct cilia development.

**Methods:**

To test the hypothesis that FOXJ1 and RFX3 cooperatively induce expression of ciliated genes in the differentiation process of basal progenitor cells toward a ciliated cell linage in the human airway epithelium, primary human airway basal cells were assessed under conditions of *in vitro* differentiation induced by plasmid-mediated gene transfer of FOXJ1 and/or RFX3. TaqMan PCR was used to quantify mRNA levels of basal, secretory, and cilia-associated genes.

**Results:**

Basal cells, when cultured in air-liquid interface, differentiated into a ciliated epithelium, expressing FOXJ1 and RFX3. Transfection of FOXJ1 into resting basal cells activated promoters and induced expression of ciliated cell genes as well as both FOXJ1 and RFX3, but not basal cell genes. Transfection of RFX3 induced expression of RFX3 but not FOXJ1, nor the expression of cilia-related genes. The combination of FOXJ1 + RFX3 enhanced ciliated gene promoter activity and mRNA expression beyond that due to FOXJ1 alone. Corroborating immunoprecipitation studies demonstrated an interaction between FOXJ1 and RFX3.

**Conclusion:**

FOXJ1 is an important regulator of cilia gene expression during ciliated cell differentiation, with RFX3 as a transcriptional co-activator to FOXJ1, helping to induce the expression of cilia genes in the process of ciliated cell differentiation of basal/progenitor cells.

## Background

Cilia are typically classified as either motile or primary [1-3]. Motile multiciliated cells typically contain 100–300 specialized cilia that are able to beat in a coordinated fashion and move liquid across the cell surface [4,5]. These cilia are found in only a few cell types in humans, including the airway epithelium lining the lung and sinuses, ependymal cells lining the ventricles of the brain and spinal canal, and the epithelium of the oviducts and epididymal ducts [5,6]. Motile cilia also occur as solitary structures, as sperm flagella, and in the embryonic node [7]. Primary cilia are nonmotile, solitary structures that are present in many cell types, and often have sensory functions such as in the retina and renal tubules [8,9].

The multiciliated cells of the human airway epithelium, accounting for 50 to 90% of the airway epithelial cell population [4,5,10,11], perform the critical function of transporting mucus in a cephalad direction to remove inhaled environmental contaminants from the airways [4,5]. Dysfunction of this transport apparatus, whether acquired or inherited, results in mucus congestion in the lower airways and serves as a nidus for recurrent infections [6,12]. The airway multiciliated cells are derived from stem/progenitor cells within the basal cell population of the airway epithelium, cells capable of replicating and differentiating into secretory, intermediate and ciliated cells [13-15]. Generation of multiciliated cells in the adult airway epithelium is an ongoing process during airway epithelium homeostasis, and in response to injury [4,5]. Substantial advances have been made in understanding the key regulators of motile and primary ciliated cell differentiation in model organisms such as mice, zebrafish and xenopus [16-20], but the transcriptional network regulating the differentiation of human airway basal progenitor cells into motile multiciliated cells is not well understood. Forkhead box J1 (FOXJ1) is one of the most well characterized transcription factors involved in motile ciliated cell differentiation in model organisms and has been suggested as a key regulator of the motile ciliated cell differentiation program [19-29]. To a variable degree, depending on the species, organ and whether multi – or monociliated, the role of FOXJ1 in the differentiation of motile ciliated cells is modulated by the transcription factor Not homeobox (NOTO) and the regulatory factor X (RFX)-family [16,20,24,30,31].

With this background, we asked two questions. First, is FOXJ1 a key regulator of the multiciliated cell differentiation program of the human airway epithelium? Second, what roles do the transcriptional regulators NOTO and/or RFX play in human airway ciliated cell differentiation? To answer these questions, we capitalized on the recent development in our laboratory to culture pure populations of primary human airway epithelial basal cells obtained by bronchoscopy and airway epithelial brushing of healthy individuals [32]. These basal cells have transcriptomes distinct from that of the airway differentiated cells, and are capable of differentiating into a multiciliated epithelium on air liquid interface cultures. The data demonstrate that in the human airway epithelium, FOXJ1 is a key regulator of multiciliated cell differentiation, that NOTO does not play a role in this process, and that RFX3 functions as transcriptional co-activator to FOXJ1, inducing expression of cilia genes involved in the differentiation towards the multiciliated cell lineage from basal progenitor cells.

## Methods

### Ethics statement

Subjects were evaluated at the Department of Genetic Medicine Clinical Research Facility under the auspices of Weill Cornell and Rockefeller University NIH Clinical Translational Science Centers, using Weill Cornell Institutional Review Board clinical protocols approved for this study. Written informed consent was obtained.

### Sampling the airway epithelium

Bronchoscopy was used to collect large airway epithelial cells by brushing the epithelium. The complete differentiated airway epithelium from 5 healthy, nonsmoking individuals was evaluated, and for 14 other individuals, the epithelium was cultured under conditions to obtain pure populations of basal cells as previously described [32-34] (see Additional file 1: Additional Methods for more detail).

### Culture and immunohistochemistry characterization of basal cells

The basal cell culture and immunohistochemistry characterization protocol have been previously described [32]. Cells were sub-cultured at day 7 to a density of 10^4^ cells/cm^2^. Cells from passage 1 to 5 were characterized and used in this study (see Additional file 1: Additional Methods for more detail).

### TaqMan quantitative real-time RT-PCR

TaqMan real-time RT-PCR was performed as previously described [35]. Relative expression levels were determined with the average value of untransfected or EGFP-control plasmid transfected basal cells as the normalizer (see Additional file 1: Additional Methods for more detail).

### Western analysis

Basal cell cultures and exogenous FOXJ1 and RFX3 expression were assessed by Western analysis as previously described [35]. Immobilized proteins were reacted with anti-KRT5; anti-KRT14; anti-MUC5AC; anti-DNAI1; anti-acetylated TUBA; anti-FOXJ1; anti-RFX3 and anti-GAPDH antibodies (see Additional file 1: Additional Methods for more detail).

### Airway epithelium differentiation in air-liquid interface culture

To assess expression profiles during the differentiation of human basal cells to ciliated cells, ciliated cell differentiation was induced from basal cells (n = 3 subjects) *in vitro* using air-liquid interface (ALI) cultures as previously described [32] (see Additional file 1: Additional Methods for more detail).

### Gene transfer to primary human airway basal cells

Human FOXJ1 and RFX3 cDNA were subcloned into expression plasmids to generate PGK.FOXJ1.IRES.EGFP, PGK.RFX3.IRES.EGFP, CMV.FLAG-FOXJ1 and CMV.FLAG-RFX3 expression plasmids. PGK.EGFP and CMV.EGFP expression plasmids were used as a control expression plasmid, respectively. Firefly luciferase (Luc) reporter gene plasmids driven by the direct upstream promoters of DNALI1, SPAG6, KRT14 and FOXJ1 were generated using standard cloning methods. DNAI1-Luc, TEKT1-Luc, RFX3-Luc, RFX2-Luc and Random sequence-Luc reporter gene plasmids were commercially available. A Renilla luciferase control reporter plasmid was used for normalization of transfection efficiency. The plasmids were transfected into primary human airway epithelial basal cells using lipofectamine LTX and promoter firefly luciferase activity was read in a luminometer. The data are reported as fold-induction (FOXJ1 compared to EGFP or FOXJ1 + RFX3 compared to FOXJ1) of at least three independent experiments read in triplicate, normalized to Renilla luciferase activity (see Additional file 1: Additional Methods for more detail).

### FOXJ1- RFX3 interaction

To assess the interaction of human FOXJ1 and RFX3, 293A cells were transfected with PGK.FOXJ1 and CMV.FLAG-RFX3 expression plasmids. Proteins were immunoprecipitated using EZview Red Anti-FLAG M2 affinity gel and eluted with FLAG peptide (see Additional file 1: Additional Methods for more detail).

### Statistical analysis

All data in this study are presented as mean ± standard error. Statistical comparisons between continuous variables were calculated using an unpaired, two-tailed t test with unequal variance. A p-value <0.05 was considered to be significant (see Additional file 1: Additional Methods for more detail).

## Results

### Primary human airway epithelial basal cell cultures

**Figure 1:**
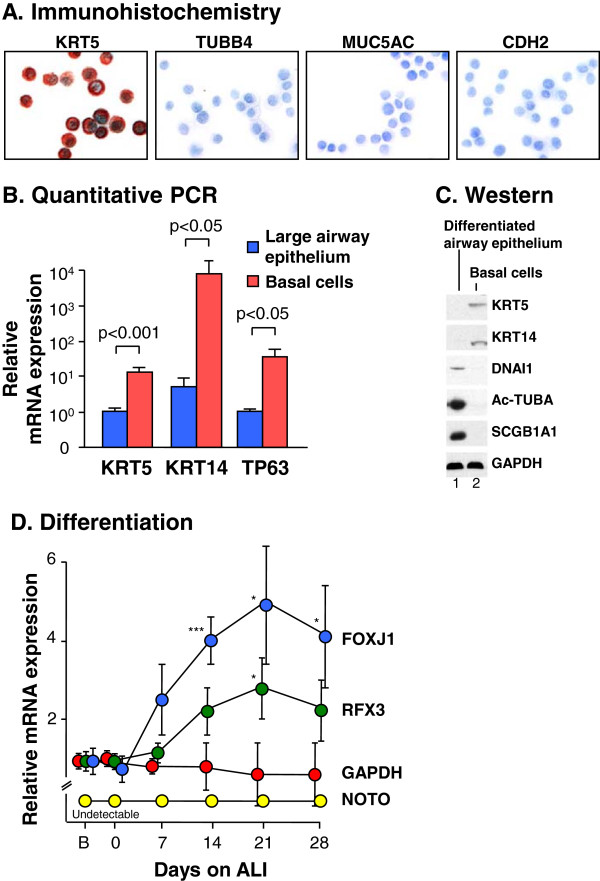
**Primary human airway epithelial basal/progenitor cell cultures. A.** Immunocytochemical verification of basal cell phenotype. Airway basal cells from healthy nonsmokers (n = 14) were purified by culturing large airway epithelial cells obtained by bronchoscopy and brushing. After 7 days of culture, the cells were trypsinized and cytospin preparations were assessed for expression of cytokeratin 5 (KRT5; basal cell-specific marker); β-tubulin IV (TUBB4; marker of ciliated cells); mucin 5AC (MUC5AC; secretory cell marker); and N-cadherin (CDH2; marker for mesenchymal cells). **B.** TaqMan quantitative real-time RT-PCR assessment of mRNA expression of the basal cell markers KRT5, KRT14 and tumor protein p63 (TP63) in complete large airway epithelial cell population (n = 5 subjects) recovered by airway brushing (blue bars) compared to basal cell cultures (day 7; red bars. n = 3 subjects). **C.** Western analysis for the basal cell markers KRT5 and KRT14, the cilia markers dynein, axonemal, intermediate chain 1 (DNAI1) and acetylated α-tubulin (Ac-TUBA), the secretory cell marker secretoglobin family 1A member 1 (SCGB1A1) and the housekeeping gene glyceraldehyde-3-phosphate dehydrogenase (GAPDH). Lane 1 – differentiated airway epithelium obtained by brushing. Lane 2 - cell extracts from basal cell cultures (day 7). **D.** Forkhead box J1 (FOXJ1), regulatory factor X3 (RFX3), Not homeobox (NOTO) and GAPDH mRNA expression during basal cell differentiation on air liquid interface (ALI) over 28 days (n = 3 subjects per time point). The expression was assessed by TaqMan quantitative real-time RT-PCR and a gene was deemed “undetectable” if not detected after 40 cycles of assessed undiluted cDNA. B = basal cells not cultured on ALI. * p < 0.05; ** p < 0.01; *** p < 0.005 compare all to GAPDH.

To test the hypothesis that FOXJ1 is a key regulator of the motile multiciliated cell differentiation program in the human airway epithelium, we first established primary human airway epithelial basal cell cultures [32]. After 7 days in culture, immunohistochemistry of cytospin preparations demonstrated the cells expressed the basal cell marker cytokeratin (KRT) 5, but did not express the ciliated cell marker β-tubulin IV, the secretory cell marker mucin 5AC or the mesenchymal cell marker N-cadherin (Figure 1A). TaqMan quantitative real-time RT-PCR assessment of the basal cell markers KRT5, KRT14 and tumor protein p63 (TP63) confirmed significantly elevated mRNA levels of these markers in basal cell cultures compared to large airway epithelium recovered by airway brushing (Figure 1B). Expression of the basal cell markers KRT5 and KRT14 and a lack of the cilia markers dynein, axonemal, intermediate chain 1 (DNAI1) and acetylated α-tubulin and the secretory cell marker secretoglobin family 1A member 1 (SCGB1A1) expression were confirmed by Western analysis of basal cell cultures compared to the complete large airway epithelial cell population (Figure 1C).

### Temporal expression of FOXJ1 and RFX3 during human airway ciliated cell differentiation

Assessment of expression of the putative cilia-associated transcriptional regulators FOXJ1, NOTO and RFX3 during human airway basal cell to ciliated cell differentiation on air liquid interface cultures (ALI) *in vitro* demonstrated that the expression pattern over time for both FOXJ1 and RFX3 was characteristic of genes participating in the ciliated cell differentiation program [36]. In contrast, NOTO, a suggested FOXJ1-upstream master regulator in mouse notochord ciliogenesis [16], was not expressed at any time-point (Figure 1D). Interestingly, NOTO was also not expressed in the complete airway epithelium, suggesting that the signaling network regulating airway epithelial motile ciliogenesis in the human airway epithelium is somewhat different than notochord ciliogenesis in mice and zebrafish (not shown).

### Effect of FOXJ1 gene transfer to primary human basal cells

**Figure 2:**
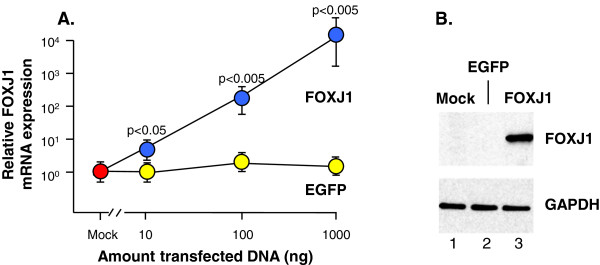
**Expression of the transcription factor FOXJ1 in primary human basal cells after transfection with a FOXJ1-expression plasmid. A.** TaqMan quantitative real-time RT-PCR assessment of the relative FOXJ1 mRNA expression in untransfected basal cells (mock), compared to increasing amounts EGFP-transfected or FOXJ1-transfected basal cells. **B.** Western analysis assessment of FOXJ1 and GAPDH expression in untransfected basal cells and cells transfected with either the control EGFP or FOXJ1 expression plasmids. Data represents the mean expression level ± standard error of pooled data from three replicated individual experiments with cells from three different subjects. All assays were carried out 24 hr after transfection.

To address the role of FOXJ1 in human airway epithelial cell differentiation, we first assessed the efficiency by which FOXJ1 expression could be induced by plasmid-mediated gene transfer of a FOXJ1-expression plasmid to primary human airway basal cells. TaqMan quantitative real-time RT-PCR demonstrated a dose-dependent relationship between the amount of transfected FOXJ1 plasmid and the mRNA levels of FOXJ1 with 10^4^-times higher FOXJ1 mRNA levels in FOXJ1 (1000 ng) expression plasmid transfected basal cells compared to untransfected (mock) or EGFP (1000 ng) transfected basal cells (Figure 2A). The transfection protocol typically yielded 55-65% transfection efficiency. Western analysis of FOXJ1 and GAPDH on untransfected basal cells and basal cells transfected with either the EGFP control or FOXJ1 expression plasmids confirmed a robust FOXJ1 expression in FOXJ1 transfected basal cells (Figure 2B).

**Figure 3:**
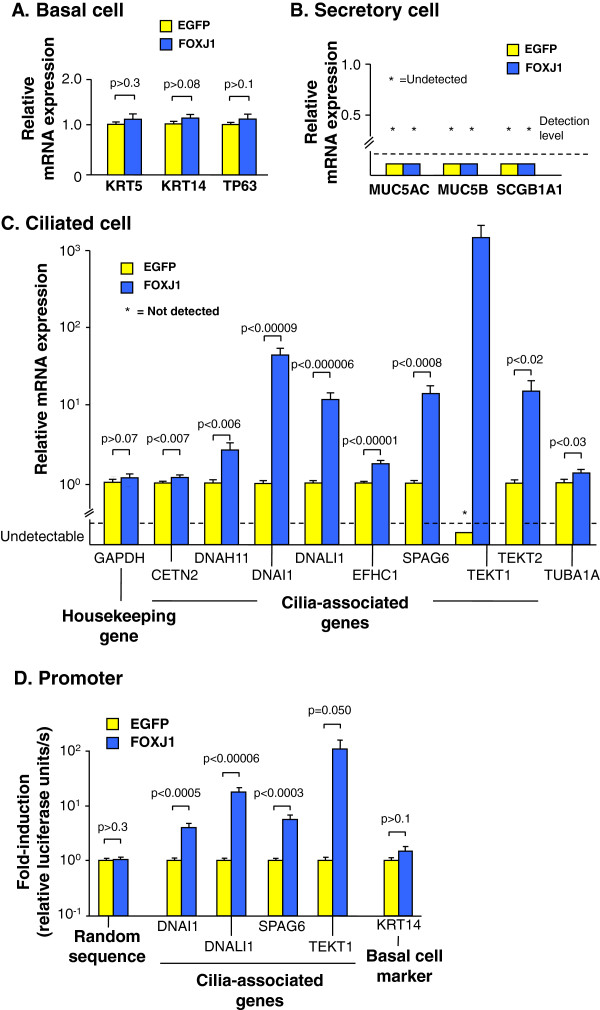
**FOXJ1-mediated expression of cilia-related genes and enhancement of their promoters in primary human basal cells transfected with either control EGFP or FOXJ1 expression plasmids.** All data is based on TaqMan quantitative real-time RT-PCR 48 hr after transfection and a gene was deemed “undetectable” if not detected after 40 cycles of assessed undiluted cDNA. **A.** Basal cell markers KRT5, KRT14 and TP63. **B.** Secretory cell markers MUC5AC, MUC5B and SCGB1A1. **C.** Ciliated cell-associated genes, including centrin 2 (CETN2); dynein, axonemal, heavy chain 11 (DNAH11), dynein, axonemal, intermediate chain 1 (DNAI1); dynein, axonemal, light intermediate chain 1 (DNALI1); EF-hand domain, C-terminal, containing 1 (EFHC1); sperm associated antigen 6 (SPAG6), tektin 1 (TEKT1), TEKT2 and tubulin, alpha 1a (TUBA1A). **D.** Shown is firefly luciferase activity in basal cells transfected with control EGFP or FOXJ1 expression plasmids together with firefly luciferase reporter gene plasmids driven by promoters of the ciliated cell-associated genes DNALI1, DNAI1, SPAG6 and TEKT1. Controls include the promoter for the basal cell marker KRT14 and a random sequence. The data were normalized for transfection efficiency per well by Renilla luciferase activity from co-transfected Renilla luciferase plasmid (pTK-RL). Bars represent mean ± standard error of pooled data from replicates of a minimum of three individual experiments with cells from three different subjects.

To investigate whether gene transfer of FOXJ1 to basal cells under conditions that normally prevent cell differentiation was sufficient to induce differentiation towards the multiciliated cell lineage, TaqMan quantitative real-time RT-PCR was used to assess EGFP or FOXJ1 expression plasmid transfection of basal cells maintained under standard basal cell culture conditions. FOXJ1 transfection had no effect on the mRNA levels of the basal cell markers KRT5, KRT14 and TP63 or expression of the secretory cell markers MUC5AC, MUC5B and SCGB1A1, not normally expressed in basal cell cultures (Figure 3A, B). In contrast, FOXJ1 expression in basal cells induced the expression of a panel of cilia-associated genes, including centrin 2 (CETN2); dynein, axonemal, heavy chain 11 (DNAH11); dynein, axonemal, intermediate chain 1 (DNAI1); dynein, axonemal, light intermediate chain 1 (DNALI1); EF-hand domain, C-terminal, containing 1 (EFHC1); sperm associated antigen 6 (SPAG6); tektin 1 (TEKT1), TEKT2 and tubulin, alpha 1a (TUBA1A; Figure 3C and Additional file 2: Table S1). Consistent with this data, co-transfection of the control EGFP or the FOXJ1 expression plasmids with plasmids carrying the firefly luciferase reporter gene driven by promoters of the ciliated cell-associated genes DNAI1, DNALI1, SPAG6 and TEKT1 demonstrated that FOXJ1 significantly induced promoter activity from the ciliated cell-associated genes while promoter activity from the basal cell specific gene KRT14 or a random sequence remained unaffected (Figure 3D). We also attempted to assess gene expression in the human samples with the available antibodies, but the antibodies did not work well, with weak staining and lack of specificity.

### Role of RFX3

**Figure 4:**
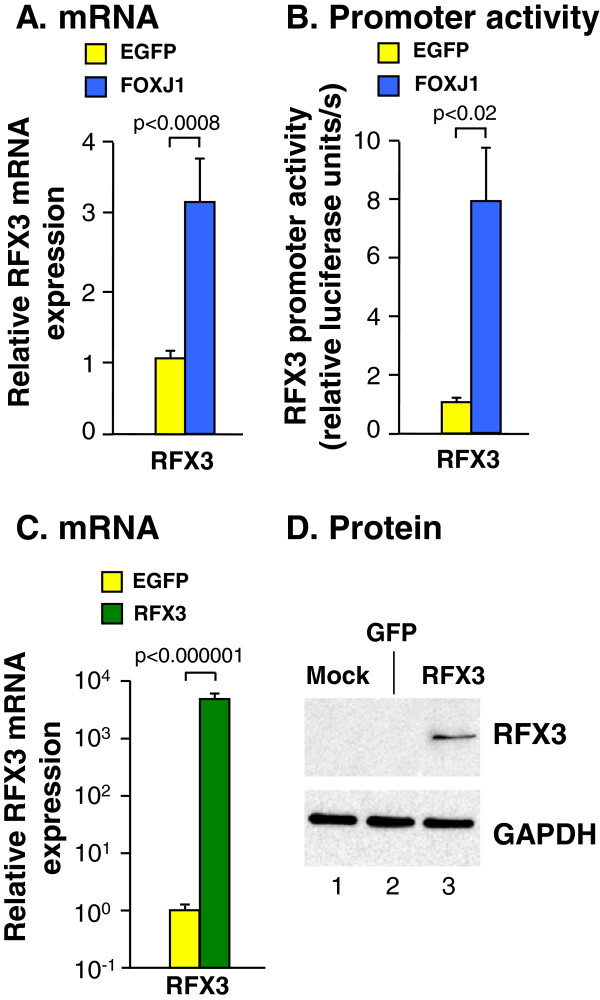
**Expression of cilia-associated transcription factor regulatory factor X3 (RFX3) in basal cells after transfection with a FOXJ1 or RFX3-expression plasmid. A.** TaqMan quantitative real-time RT-PCR assessment of the relative endogenous RFX3 mRNA expression in EGFP or FOXJ1-transfected basal cells 48 hr after transfection. **B.** Firefly luciferase activity in basal cells transfected with control EGFP or FOXJ1 expression plasmids together with firefly luciferase reporter gene plasmid driven by the RFX3 promoter 48 hr after transfection. **C.** TaqMan quantitative real-time RT-PCR assessment of the relative RFX3 mRNA expression in basal cells transfected with the control EGFP or RFX3 expression plasmid. **D.** Western analysis assessment of RFX3 and GAPDH expression in untransfected basal cells and cells transfected with either control EGFP or RFX3 expression plasmid. Data represents the mean ± standard error of pooled data from replicates of a minimum of three individual experiments with cells from three different subjects.

To investigate the role of cilia-associated transcriptional regulator RFX3 in human airway epithelial motile ciliogenesis, TaqMan quantitative real-time RT-PCR was used to assess RFX3 expression in basal cells transfected with FOXJ1 expression plasmid and RFX3 promoter activity was assessed by co-transfection with a plasmid carrying the firefly luciferase reporter gene driven by the RFX3 promoter (Figure 4A, B). The data demonstrated increased RFX3 promoter activity and mRNA expression in cells transfected with the FOXJ1 expression plasmid compared to cells transfected with the control EGFP plasmid, suggesting a role for RFX3 downstream of FOXJ1 in human airway epithelial ciliogenesis. To investigate whether RFX3 could, like FOXJ1, induce differentiation from the basal cell lineage towards the multiciliated cell lineage, a RFX3 expressing plasmid was transfected into the basal cells. While RFX3 expression was demonstrated by TaqMan quantitative real-time RT-PCR and Western analysis in basal cells transfected with a RFX3 expression plasmid (Figure 4C, D), none of the wide panel of FOXJ1-induced cilia associated genes or the basal and secretory cell markers were induced in RFX3 transfected basal cells (Additional file 3: Figure S1A-C). These data suggest that although FOXJ1 induces RFX3 expression in human airway epithelial cells, RFX3 expression alone is not sufficient to drive the human basal cell to multiciliated cell differentiation process.

### RFX3 acts as a transcriptional co-activator to FOXJ1 that enhances the expression of cilia-associated genes

**Figure 5:**
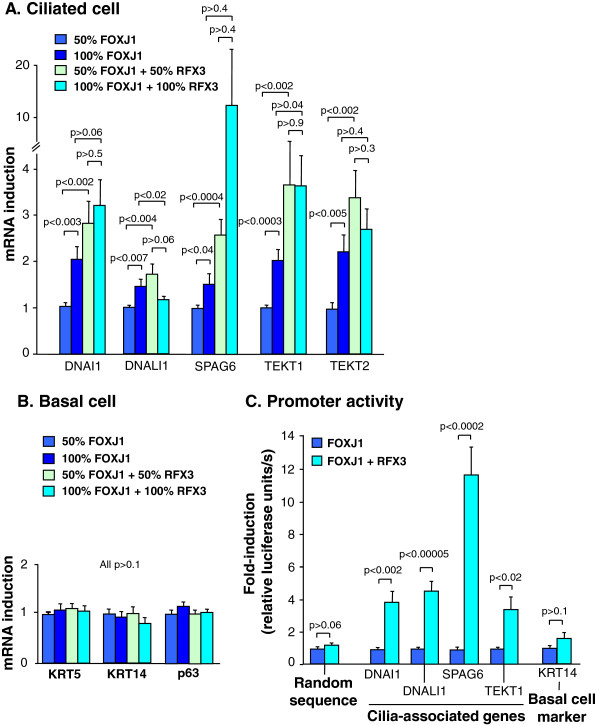
**RFX3-mediated enhancement of FOXJ1-induced expression of ciliated cell-associated genes in primary human basal cells transfected with both a FOXJ1 and RFX3 expression plasmid. A.** TaqMan quantitative real-time RT-PCR assessment of the relative mRNA expression of the ciliated cell-associated genes DNAI1, DNALI1, SPAG6, TEKT1 and TEKT2 in basal cells transfected with 50% and 100% FOXJ1 expression plasmids compared to basal cells co-transfected with 50% and 100% of FOXJ1 and RFX3 expression plasmids together for 48 hr after transfection. **B.** TaqMan quantitative real-time RT-PCR assessment of the basal cell markers KRT5, KRT14 and TP63. **C.** Firefly luciferase activity assessment of basal cells transfected with firefly luciferase reporter gene plasmids driven by promoters of the ciliated cell-associated genes DNALI1, DNAI1, SPAG6 and TEKT1; or the basal cell marker KRT14; or a random sequence as a negative control. Data were normalized for transfection efficiency per well by Renilla luciferase activity from co-transfected Renilla luciferase plasmid (pTK-RL) 48 hr after transfection. Bars represent mean ± standard error of pooled data from replicates of a minimum of three individual experiments from different subjects.

RFX3 has an established role in the ciliogenetic process in animal models [16,17,20,24,30], and although RFX3 alone does not seem to induce human airway epithelial differentiation (Additional file 3: Figure S1A-C), we questioned whether RFX3 could play a role in human airway ciliogenesis by mediating FOXJ1-induced multiciliated cell differentiation. Interestingly, RFX3 significantly enhanced the FOXJ1-induced mRNA expression of the ciliated cell associated genes DNAI1, DNALI1, SPAG6, TEKT1 and TEKT2 (all p < 0.004) in basal cells transfected with FOXJ1 and RFX3 expression plasmids together compared to basal cells transfected with FOXJ1 and EGFP expression plasmids together (Figure 5A). In addition, the expression of these cilia associated genes were induced in a dose-dependent manner by FOXJ1 (all p < 0.04) in basal cells transfected with an increasing dose of FOXJ1 expression plasmid (Figure 5A), demonstrating a tight FOXJ1-RFX3 mediated regulation of these genes. Expression of the basal cell markers KRT5, KRT14 and TP63 remained unaffected (Figure 5B). Consistent with this data, promoter activity of the cilia-associated genes DNAI1, DNALI1, SPAG6 and TEKT1 was enhanced (p < 0.02) in basal cells transfected with FOXJ1 and RFX3 together compared to cells transfected with FOXJ1 and EGFP together when co-transfected with the respective firefly luciferase reporter plasmids (Figure 5C). Promoter activity of the basal cell marker KRT14 and a random sequence were unaffected by FOXJ1 and RFX3 co-expression (Figure 5C). These data suggest that RFX3 alone is not sufficient to induce human airway basal cells to differentiate to multiciliated cells, but rather acts as a transcriptional co-activator to FOXJ1 that magnifies the induction of cilia associated genes.

### Human RFX3 interaction with human FOXJ1

**Figure 6:**
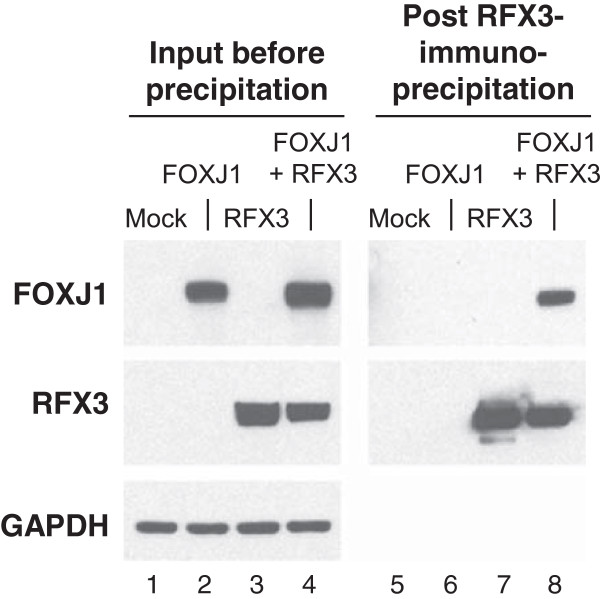
**Human FOXJ1 and RFX3 protein-to-protein interaction.** 293A cells were transfected with FOXJ1 and RFX3 expression plasmids. After 48 hr, cell lysates were prepared in lysis buffer and incubated overnight with Anti-FLAG M2 affinity gel. Input and immunoprecipitated fractions were resolved by SDS-PAGE and analyzed by Western blotting with antibody specific for FOXJ1, FLAG and GAPDH as a loading control. Lanes 1-4 - Before immunoprecipitation (input). Lanes 5-8 - After RFX3 targeted immunoprecipitation. Lane 1 - PGK.EGFP and CMV.empty (mock); lane 2 - PGK.FOXJ1 and CMV.empty (FOXJ1); lane 3 - CMV.FLAG-RFX3 and PGK.EGFP (RFX3); lane 4 - PGK.FOXJ1 and CMV.FLAG-RFX3 (FOXJ1 + RFX3); lane 5 - PGK.EGFP and CMV.empty (mock); lane 6 - PGK.FOXJ1 and CMV.empty (FOXJ1); lane 7 - CMV.FLAG-RFX3 and PGK.EGFP (RFX3); and lane 8 - PGK.FOXJ1 and CMV.FLAG-RFX3 (FOXJ1 + RFX3).

Western analysis was used to address whether RFX3 interacts with FOXJ1 via protein-to-protein interaction. Immunoprecipitation with anti-FLAG affinity beads of functionally intact (not shown) FLAG-tagged RFX3 co-precipitated FOXJ1 in 293A cells transfected with FLAG-RFX3 expression plasmids together with FOXJ1 expression plasmids, compared to mock, FOXJ1, or FLAG-RFX3 alone transfected cells (Figure 6). Analysis of input lysates before immunoprecipitation using antibodies against FOXJ1 and RFX3 revealed robust expression of each in the appropriate lysate. Detection of GAPDH confirmed an equal amount of total protein in each (Figure 6). These data demonstrate that RFX3 can interact and function as a co-activator to FOXJ1 via direct protein-to-protein interaction between the two factors or indirect interaction within an intracellular protein complex.

### Auto-regulatory feed-back mechanism of FOXJ1 and RFX3

**Figure 7:**
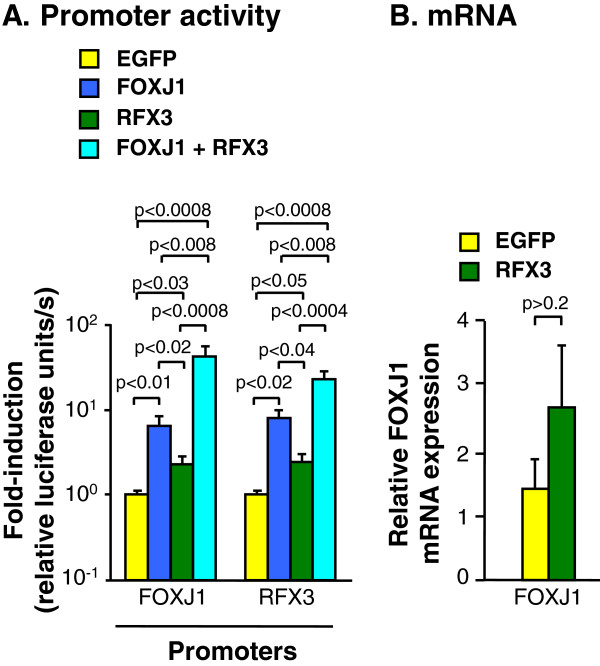
**FOXJ1-RFX3 auto-regulatory feedback. A.** Firefly luciferase activity in basal cells transfected with control EGFP, FOXJ1, RFX3 or FOXJ1+ RFX3 expression plasmids together with firefly luciferase reporter gene plasmids driven by the FOXJ1 and RFX3 promoters. The data were normalized for transfection efficiency per well by Renilla luciferase activity from co-transfected Renilla luciferase plasmid (pTK-RL) 48 hour after transfection. Bars represent mean ± standard error of pooled data from a minimum of three individual experiments assessed for 48 hr after transfection. **B.** TaqMan quantitative real-time RT-PCR assessment of the relative endogenous FOXJ1 mRNA expression in EGFP or RFX3-transfected basal cells for 48 hr after transfection. Bars represent mean ± standard error of pooled data from replicates of three individual experiments from different subjects.

To address whether or not FOXJ1 and RFX3 expression is self-regulated in human airway epithelial cell differentiation, we assessed the RFX3 and FOXJ1 promoter activity in basal cells transfected with FOXJ1 and/or RFX3 expression plasmid and co-transfection with plasmids carrying the firefly luciferase reporter gene driven by either the FOXJ1 or the RFX3 promoter (Figure 7A). Corroborating the FOXJ1 induced RFX3 mRNA expression (Figure 4A), both FOXJ1 and RFX3 promoter activity were induced 6.0- (p < 0.01) and 7.9-fold (p < 0.02) in basal cells transfected with FOXJ1 expression plasmid compared to cells transfected with the control EGFP expression plasmid. RFX3 co-expression further enhanced the FOXJ1-induced activity of the FOXJ1 promoter 2.9-fold (p < 0.008) and the RFX3 promoters 2.7-fold (p < 0.008). In contrast to the cilia-associated genes, RFX3 expression was sufficient to induce both FOXJ1 2.2-fold (p < 0.008) and RFX3 promoter activity 2.4-fold (p < 0.05) when expressed alone, although at lower levels than FOXJ1. RFX3 did not, however, significantly induce endogenous FOXJ1 mRNA expression in basal cells transfected with RFX3 expression plasmid alone (Figure 7B). In summary, these data suggest that both FOXJ1 and RFX3 can regulate their own expression to different degrees and that FOXJ1 is the major inducer of a positive auto-regulatory feedback mechanism where FOXJ1 is upstream of RFX3.

### Role of RFX2

RFX-family members other than RFX3 have also been implicated in cilia homeostasis in various model organisms [18,20,26,37]. Assessment of expression of the different RFX-transcription factor members during human airway basal cell to ciliated cell differentiation on air liquid interface cultures *in vitro* demonstrated that the expression patterns over time for RFX2 were also characteristic of genes participating in the ciliated cell differentiation program [36], in similarity with FOXJ1 and RFX3 (Figure 1D, Additional file 4: Figure S2A). We further assessed the ability of FOXJ1 and /or RFX3 to induce endogenous RFX2 mRNA expression in basal cells transfected with FOXJ1 and /or RFX3 expression plasmids, as well as induced RFX2 promoter activity in basal cells co-transfected with FOXJ1 and /or RFX3 expression plasmids and a plasmid carrying the firefly luciferase reporter gene driven by the RFX2 promoter (Additional file 4: Figure S2B,C). The data demonstrated increased RFX2 promoter activity (p < 0.0005) and mRNA expression (p < 0.04) in cells transfected with the FOXJ1 expression plasmid alone and no significant induction of either RFX2 promoter activity or mRNA expression in cells transfected with RFX3 alone, compared to cells transfected with the control EGFP plasmid. Cells transfected with RFX3 seemed to have elevated RFX2 mRNA expression, although to a variable degree and not significant. When RFX3 was co-transfected with FOXJ1, however, the FOXJ1 induced RFX2 promoter activity (p < 0.03) was further enhanced, suggesting a similar and potentially partially overlapping role for RFX2 and RFX3 downstream of FOXJ1 in human airway epithelial ciliogenesis.

## Discussion

Motile multiciliated cells, the dominant cell type in the airway epithelium, perform the critical function of transporting mucus and entrapped inhaled environmental contaminants up from the airways [4,5]. Dysfunction of this mucociliary clearance apparatus during development or after injury results in airway mucus-plugging which constitutes a source of recurring infections. In this study, we hypothesized that in the human airway epithelium, the two transcription factors FOXJ1 and RFX3 cooperatively regulate the differentiation of cells from the basal progenitor cell lineage toward the ciliated cell linage.

Primary human airway basal cells from healthy nonsmokers induced expression of FOXJ1 and RFX3 while differentiating into a ciliated epithelium in air-liquid interface cultures. Expression of FOXJ1 in basal cells transfected and cultured on plastic induced promoter-activity from ciliated cell genes, but not basal cell gene promoters, as well as increased expression of RFX3 and a wide variety of ciliated cell genes, but not the expression of basal or secretory cell genes. In contrast, RFX3 induced FOXJ1 expression but did not induce expression of cilia-related genes, which suggest that a threshold amount of FOXJ1 is required to induce cilia gene expression. When RFX3 was expressed together with FOXJ1, however, the combination of the two factors up-regulated ciliated gene promoters and mRNA expression to a greater extent than FOXJ1 alone. Knockdown and chromatin immunoprecipitation (ChIP) experiments validating the effects of FOXJ1 and RFX3 on downstream cilia-associated genes would have been valuable, but due to technical limitations, including a baseline expression of the downstream target genes bordering or below detectable levels in basal cells, this was not feasible. A bioinformatic assessment of potential FOXJ1 and RFX3 binding sites in the promoters of the cilia-related target genes could not fully explain the FOXJ1 and RFX3 mediated transcriptional regulation of these genes (data not shown).

However, immunoprecipitation studies in 293A cells corroborated our findings and demonstrate an interaction between the human FOXJ1 and RFX3 proteins. Our data further suggest a positive auto-regulatory FOXJ1-RFX3 feedback mechanism where FOXJ1 is upstream of RFX3. Hence, our findings demonstrate that FOXJ1 is an important regulator of cilia-associated gene expression in primary human airway epithelial basal cells and that RFX3 acts as a co-activator to FOXJ1 in this process during basal/progenitor cell differentiation toward the multiciliated cell lineage.

### Ciliated cell differentiation in model organs

Much of our knowledge of motile monocilium and multiciliated cell differentiation is based on studies of the mono motile ciliated cells of the mouse node and its homologues in zebrafish and xenopus [16-19]. FOXJ1 is one of the most well characterized transcription factors involved in motile ciliated cell differentiation, where FOXJ1 is known to regulate programs promoting basal body docking and axoneme formation [19-29]. Also the RFX-family of transcription factors has been shown to be a major regulator of ciliogenesis in multiple organisms, controlling the expression of the many essential genes required for making cilia [38-42] and has recently been shown to co-regulate cilia gene expression together with FOXJ1 in Drosophila [31]. Beckers et al [16] demonstrated evidence that the murine nothochord motile monocilium differentiation program is regulated by the transcription factor NOTO, acting upstream of both FOXJ1 and RFX3. The novel nuclear protein multicilin was also recently shown to drive the expression of both FOXJ1 and multiciliated cell specific genes in frog and mouse multiciliated epithelia [43]. Several investigators, using murine models and transformed human cell lines, have demonstrated that FOXJ1 is a key regulator of airway epithelial ciliated cell differentiation [21,22,27,29,44-50]. In the airway epithelium, Notch signaling had been demonstrated to be the most upstream cue for transcriptional events during the differentiation of multiciliated and other airway epithelial cell types [41].

Murine models of airway development and cellular differentiation have been critical to our basic understanding of the mucociliary clearance apparatus during development or after injury [21,51], but the human airways differ from those of mice to some extent [13]. The networks regulating cell commitment in the airway epithelium in humans are therefore likely to differ somewhat from those of mice and other animal models.

### Human airway epithelial multiciliated cell differentiation

The human FOXJ1 gene was first cloned over 10 yr ago [52,53], yet the function of FOXJ1 in the human airway epithelium remains relatively unknown. Studies have addressed whether mutations in the FOXJ1 gene could be linked to primary ciliary dyskinesia [54,55], although this hypothesis has not been supported. The activity of the human FOXJ1 promoter has been demonstrated to be specific to ciliated cells in the mouse airway epithelium *in vivo*[56]. The mouse FOXJ1 promoter has been shown to be active in primary human airway epithelial cells and the human airway epithelial cell line H441, but not in the human fibroblast cell line HT1080 [57]. The murine and human FOXJ1 gene have been transfected into the transformed human airway epithelial BEAS-2B cell line and in the human embryonic kidney (HEK-293) cell line [21,45,57,58]. Expression of murine FOXJ1 in BEAS-2B cells failed to induce cilia development [21], but consistent with the findings in the present study, murine FOXJ1 induced SPAG6 promoter activity in these cells [58].

FOXJ1 and RFX3 are highly expressed in the small airway epithelium [32], consistent with the concept that FOXJ1 and RFX3 together may play a pivotal role in maintaining homeostasis of the multiciliated cell population in the adult human airway epithelium. In the current study, we found that NOTO is not expressed in the human airway epithelium nor is its expression induced by ALI-mediated airway epithelial differentiation *in vitro*. Hence, in contrast to murine notochord ciliogenesis, FOXJ1 and RFX3 are not regulated upstream by NOTO in the human airway epithelium. We can only speculate on the identity of these players, but possible candidates include Notch, multicilin, FOXA2 and SOX2 [41,43,59-61].

## Conclusion

Most evidence suggests that the basal cells of the human airway epithelium contain the pluripotent progenitor cell population, capable of replicating and differentiating into intermediate, secretory, and multiciliated cells [13-15,32]. The present study demonstrates that, under conditions that normally prevent cellular differentiation, FOXJ1 ± RFX3 plays a significant role in modulating differentiation steps in multiciliated cell differentiation from the basal cell lineage. These findings also support the concept that airway epithelial basal cells contain the progenitor cell population capable of differentiating into multiciliated cells.

## Competing interests

The authors declare that they have no competing interests.

## Authors’ contributions

LD carried out the cell and molecular genetic studies, performed the statistical analysis and drafted the manuscript. RKZ carried out cell studies. IC cloned reporter gene vectors. MSW carried out the IP studies. RW carried out the ALI assays. NRH participated in the design of the study. RGC participated in the design of the study, supervised the research and helped to draft the manuscript. All authors read and approved the final manuscript.

## Supplementary Material

Additional file 1 **Additional Methods.** Sampling the Airway Epithelium. Click here for file

Additional file 2: Table S1 Ciliated Cell-associated Genes. Click here for file

Additional file 3: Figure S1 Expression of cilia-related genes in primary human basal cells transfected with either control EGFP or RFX3 expression plasmids. All data is based on TaqMan quantitative real-time RT-PCR 48 hr after transfection. A. Ciliated cell-associated genes, including CETN2, DNAH11, DNAI1, DNALI1, EFHC1, SPAG6, TEKT1, TEKT2, and TUBA1A. B. Basal cell markers KRT5, KRT14 and TP63. C. Secretory cell markers MUC5AC, MUC5B and SCGB1A1. Bars represent mean ± standard error of pooled data from replicates of a minimum of three individual experiments with cells from different subjects assessed 48 hr after transfection. Click here for file

Additional file 4: Figure S2 RFX2 in human basal cell differentiation into ciliated cells. Shown is the temporal expression of RFX2 during basal to ciliated cell differentiation on ALI and the RFX2 mRNA expression and promoter activity in basal cells transfected with FOXJ1 and / or RFX3. A. RFX2 mRNA expression during basal cell differentiation on ALI over 28 days (n=3 per time point). The data was generated by TaqMan quantitative real-time RT-PCR analysis * p<0.05 compared to GAPDH. B. TaqMan quantitative real-time RT-PCR assessment of the relative RFX2 mRNA expression in basal cells transfected with control EGFP, FOXJ1, RFX3 or FOXJ1+ RFX3 expression plasmids. C. Firefly luciferase activity in basal cells transfected with control EGFP, FOXJ1, RFX3 or FOXJ1+ RFX3 expression plasmids together with a firefly luciferase reporter gene plasmid driven by the RFX2 promoter. The data were normalized for transfection efficiency per well by Renilla luciferase activity from co-transfected Renilla luciferase plasmid (pTK-RL). Bars represent mean ± standard error of pooled data from replicates of three individual experiments with cells from different subjects assessed 48 hr after transfection. Click here for file
