# Supplementary material for: RFX3 Modulation of FOXJ1 regulation of cilia genes in the human airway epithelium
Source: Respir Res. 2013 Jul 3;14(1):70. doi: 10.1186/1465-9921-14-70 (PMC3710277; doi:10.1186/1465-9921-14-70)
Supplement: Additional file 1 — Additional Methods. Sampling the Airway Epithelium. [file 1465-9921-14-70-S1.pdf]

Additional File 1: Additional Methods

Sampling the Airway Epithelium

Healthy, nonsmoking subjects were evaluated at the Department of Genetic Medicine Clinical Research Facility under the auspices of Weill Cornell and Rockefeller University NIH Clinical Translational Science Centers, using Institutional Review Board-approved clinical protocols. For 5 individuals, the complete differentiated airway epithelium was evaluated and for 14 other individuals, the epithelium was cultured under conditions to obtain pure populations of basal cells. All subjects were confirmed to be nonsmokers by urine levels of nicotine (<2 ng/ml) and cotinine (<5 ng/ml) with normal pulmonary function tests and chest X-ray. After obtaining written informed consent, flexible bronchoscopy was used to collect large airway epithelial cells by brushing the epithelium as previously described [1-3]. Cells were detached from the brush by flicking into 5 ml of ice-cold Bronchial Epithelium Basal Medium (BEGM, Lonza, Basel, Switzerland). An aliquot of 0.5 ml was used for differential cell count. The remainder (4.5 ml) was processed immediately for either immediate RNA or protein extraction or basal cell culture followed by RNA or protein extraction or culture on ALI followed by RNA extraction. The number of cells recovered by brushing was determined by counting on a hemocytometer. To quantify the percentage of epithelial and inflammatory cells and the proportions of basal, ciliated, secretory and intermediate cells recovered, cells were prepared by centrifugation (Cytospin 11, Shandon Instruments, Pittsburgh, PA) and stained with Diff-Quik (Baxter Healthcare, Miami, FL). In all samples the epithelial cells represented >97% of the cell population; the proportions of epithelial cells were as previously reported [3].

Culture of Basal Cells

The basal cell culture protocol utilized is detailed in Hackett et al [3]. Briefly, airway epithelial cells collected by brushing were pelleted by centrifugation (250 x g, 5 min) and

disaggregated by resuspension in 0.05% trypsin-ethylenediaminetetraacetic acid (EDTA) for 5 min, 37°C. Trypsinization was stopped by addition of HEPES buffered saline (Lonza, Basel, Switzerland) supplemented with 15% fetal bovine serum (FBS; GIBCO-Invitrogen, Carlsbad, CA), and the cells were again pelleted at 250 x g, 5 min. The pellet was resuspended with 5 ml of phosphate buffered saline, pH 7.4 (PBS), 23°C, then centrifuged at 250 x g, 5 min. Following centrifugation, the PBS was removed, the cells resuspended in 5 ml of BEGM and 5 x 10^5 cells were cultured in T25 flasks in BEGM (Lonza, Basel, Switzerland), supplemented with growth factors according to the manufacturer's instructions. The antibiotics supplied by the manufacturer of BEGM were replaced with gentamicin (50 µg/ml; Sigma, St Louis, MO), amphotericin B (1.25 µg/ml; Invitrogen, Carlsbad, CA), and penicillin-streptomycin (50 µg/ml; Invitrogen, Carlsbad, CA). Cultures were maintained in a humidified atmosphere of 5% CO_2, 37°C. Unattached cells were removed by changing medium after 12 hr. Thereafter, media was changed every 2 days and cells were sub-cultured at day 7 and thereafter when they reached approximately 80% confluence to a density of 10^4 cells/cm^2. Cells from passage 1 to 5 were characterized and used in this study.

Immunohistochemistry Characterization of Basal Cells

To characterize the basal cell cultures by immunohistochemistry, cells were trypsinized, and cytospin slide preparations were fixed in 4% paraformaldehyde for 15 min. Antigen recovery was carried out by steaming the samples for 15 min in citrate buffer solution (Labvision, Fremont, CA) followed by cooling at 23°C, 20 min. Endogenous peroxidase activity was quenched using 0.3% H_2O_2, and normal serum matched secondary antibody was used for 20 min to reduce background staining. Samples were incubated overnight at 4°C with primary antibodies, including rabbit polyclonal anti-human cytokeratin 5 antibody (1/50; Thermo Scientific, Rockford, IL) as a basal cell marker; mouse monoclonal anti-human N-cadherin

antibody (1/2500; Invitrogen, Carlsbad, CA) for mesenchymal cells; mouse monoclonal anti-human mucin 5AC antibody (1/50; Vector Laboratories, Burlingame, CA) for secretory cells; and mouse monoclonal anti-human β-tubulin IV antibody (1/2000 dilution; Biogenex, San Ramon, CA) for ciliated cells. Isotype matched IgG (Jackson Immunoresearch Laboratories, Inc, West Grove, PA) was the negative control. Vectastain Elite ABC kit and AEC substrate kit (Dako North America, Inc, Carpinteria, CA) were used to visualize antibody binding. The sections were counterstained with Mayer's hematoxylin (Polysciences, Inc, Warrington, PA) and mounted using Faramount mounting medium (Dako North America). Brightfield microscopy was done using a Nikon Microphot microscope equipped with a Plan x40 numerical aperture (NA) 0.70 objective lens. Images were captured with an Olympus DP70 CCD camera.

TaqMan Quantitative Real-time RT-PCR

TaqMan real-time RT-PCR was performed on RNA samples from the large airway epithelium of healthy nonsmokers as well as on transfected and untransfected basal cells. cDNA was synthesized from 1 to 2 μg RNA in a 50 μl reaction volume, using the TaqMan Reverse Transcriptase Reaction Kit (Applied Biosystems, Foster City, CA), with random hexamers as primers and duplicate wells were run for each sample. TaqMan PCR reactions were carried out using pre-made gene expression kits from Applied Biosystems for all target genes. NOTO expression was assessed using two different probe sets. The endogenous control was human 18S rRNA (Applied Biosystems). Relative expression levels were determined using the ddCt method, with the average value of untransfected or EGFP-control plasmid transfected basal cells as the normalizer [4]. The PCR reactions were run in an Applied Biosystems Sequence Detection System 7500, and the relative quantity was determined using the algorithm provided by the manufacturer.

Western Analysis

Basal cell cultures and exogenous FOXJ1 and RFX3 expression were assessed by Western analysis. Cells were trypsinized and lysed in radioimmunoprecipitation lysis (RIPA) buffer plus Complete Protease Inhibitor Cocktail (Roche, Mannheim, Germany) and dithiothreitol (DTT), and incubated on ice for 30 min. Lysates were clarified by centrifugation at 22,500 x *g* for 10 min in an Eppendorf 5415C microcentrifuge at 4°C. Total protein concentration was measured using the Bio-Rad (Hercules, CA) protein assay to the manufacturer's guidelines. For samples of large airway epithelium, cells were obtained directly from brushings and, following two washes with PBS, processed in an identical manner to the cultured basal cells. NuPAGE® LDS Sample Buffer (4X) supplemented with NuPAGE® reducing buffer (10X) was added to each sample before boiling for 10 min and SDS-polyacrylamide gel electrophoresis (PAGE) analysis using NuPAGE® 4 to 12% Bis-Tris gradient gels (Invitrogen). Proteins were transferred onto nitrocellulose membranes with a Bio-Rad Semi-Dry apparatus before Western analysis. After blocking membranes overnight at 4°C in 4% nonfat milk in PBS containing 0.1% Tween-20 (PBST), immobilized proteins were reacted with cell type specific antibodies in 4% nonfat milk in PBST for 1 hr, 23°C with shaking, including: rabbit polyclonal anti-human cytokeratin 5 (1/3000; Thermo Scientific); mouse monoclonal anti-human cytokeratin 14 (1/3000; R&D Biosystems, Minneapolis, MN); mouse monoclonal anti-human mucin 5AC (1/500; Vector Laboratories, Burlingame, CA); rabbit polyclonal anti-human dynein intermediate chain 1 (DNAI1; 1/3000; Sigma, St Louis, MO) and mouse anti-acetylated alpha tubulin (Ac-TUBA, 1/ 2000, Sigma). FOXJ1 and RFX3 expression were detected with mouse monoclonal anti-FOXJ1 (e-Bioscience, San Diego, CA) and mouse anti-RFX3 (Novus Biologicals, Littleton, CO). Mouse monoclonal anti-human glyceraldehyde dehydrogenase (GAPDH; 1/5000; Santa Cruz Biotechnology, Inc.) was used as a loading

control. Following the primary antibody incubation, membranes were washed three times for 5 min each with PBST, incubated with an anti-rabbit or anti-mouse antibody conjugated to horseradish peroxidase in 4% nonfat milk in PBST for 1 hr, 23°C with shaking. Upon completion of secondary antibody incubation, the membranes were washed again three times for 5 min with PBST and twice with PBS, and antibodies were visualized after the addition of ECL Western Blotting Detection Reagents (GE Healthcare Biosciences, Pittsburgh, PA) by exposure to X-ray film.

Airway Epithelium Differentiation in Air-liquid Interface Culture

To determine whether FOXJ1 and/or the RFX-transcription factor family are expressed during the differentiation of human basal cells to ciliated cells, ciliated cell differentiation was induced from basal cells (n=3 subjects) *in vitro* using air-liquid interface (ALI) cultures [3]. The basal cells were trypsinized after 7 days of culture and seeded at a density of 6 x 10^5 cells/cm^2 onto a 0.4 μm pore-sized Costar Transwells inserts (Corning Incorporated, Corning, NY) pre-coated with type IV collagen (Sigma, St Louis, MO). The initial culture medium consisted of a 1:1 mixture of DMEM and Ham's F-12 medium (GIBCO-Invitrogen, Carlsbad, CA) containing 100 U/ml penicillin, 5% fetal bovine serum 100 μg/ml streptomycin, 0.1% gentamicin, and 0.5% amphotericin B. On the next day, the medium was changed to 1:1 DMEM/Ham's F12 (including antibiotics described above) with 2% Ultroser G serum substitute (BioSerpa S.A., Cergy-Saint-Christophe, France). Following 2 days of culturing on the membrane, the media were removed from the upper chamber to expose the apical surface to air and establish the ALI (referred to as ALI "day 0"). The cells were then grown at 37°C, 8% CO_2, and the culture medium was changed every other day. Following 5 days on ALI, the cells were grown at 37°C, 5% CO_2 until harvested. RNA from one well of each subject were harvested at basal cell culture day 7 and ALI day 0, 7, 14, 21 and 28.

RNA Processing

Total RNA was extracted using a modified version of the TRIzol method (Invitrogen, Carlsbad, CA). RNA was purified directly from the aqueous phase (RNeasy MinElute RNA purification kit, Qiagen, Valencia, CA). RNA samples were stored in RNA Secure (Ambion, Austin, TX) at -80°C. RNA integrity was determined by assessing an aliquot of each RNA sample on an Agilent Bioanalyzer (Agilent Technologies, Palo Alto, CA). The concentration was determined using a NanoDrop ND-1000 spectrophotometer (NanoDrop Technologies, Wilmington, DE). Double-stranded cDNA was synthesized from 1 μg total RNA using the GeneChip One-Cycle cDNA Synthesis Kit.

Gene Transfer to Primary Human Airway Basal Cells

In order to assess the function of FOXJ1 and RFX3 in basal cells, human FOXJ1 and RFX3 cDNA were subcloned using standard methods into the multiple cloning site (MCS) of a PGK.MCS.IRES.EGFP expression plasmid driven by the phosphoglycerate kinase (PGK) promoter which co-expressed enhanced green fluorescent protein (EGFP) via an internal ribosome entry site (IRES) [5]. A PGK.EGFP expression plasmid where the PGK promoter directly drives EGFP expression was used as a control expression plasmid [6]. FOXJ1 and RFX3 cDNA were also subcloned in the cytomegalovirus (CMV)-promoter driven 5’ FLAG-tag expression vector, pCMV-Tag2B (Agilent Technologies, Santa Clara, CA), to generate CMV.FLAG-FOXJ1 and CMV.FLAG-RFX3 expression plasmids, and a CMV.EGFP expression plasmid was used as a control expression plasmid. Both mRNA and promoter activity of ciliated cell-associated genes were induced to comparable levels using either PGK or CMV driven FOXJ1 and RFX3 expression (not shown). Firefly luciferase (Luc) reporter gene plasmids driven by the direct upstream promoters of the ciliated cell-associated genes DNALI1 (0.7 kb) and SPAG6 (1.7 kb), the basal cell marker KRT14 (2.6 kb) and the transcription factor FOXJ1 (1.3

kb) were cloned into the TAL-Luc plasmid (Clontech Laboratories, Inc., Mountain View, CA) after removal of the minimal TATA-box using standard methods. A commercially available firefly luciferase reporter gene plasmid driven by the direct upstream promoter (1 kb) of the ciliated cell associated genes DNAI1 and TEKT1, the transcription factors RFX3 and RFX2, as well as a negative control random sequence reporter gene plasmid (SwitchGear Genomics, Menlo Park, CA) were used. A Renilla luciferase control reporter plasmid (pRL-TK, Promega, Madison, WI) was used for normalization of transfection efficiency.

The plasmids were transfected into primary human airway epithelial basal cells seeded at approximately $8x10^4$ cells/24-well in a cell culture plate the day before transfection using 1 μg lipofectamine LTX, 0.5 μl PLUS-reagent (Invitrogen, Carlsbad, CA) and 0.5 μg of total plasmids diluted in 100 μl BEBM media (Lonza). To quantify promoter function, cells were lysed in Dual Luciferase Buffer (Promega, Madison, WI) and promoter firefly luciferase activity was read in a luminometer (Berthold detection systems, Pforzheim, Germany). The data are reported as fold-induction (FOXJ1 compared to EGFP or FOXJ1+RFX3 compared to FOXJ1) of at least three independent experiments read in triplicate, normalized to Renilla luciferase activity.

FOXJ1- RFX3 Interaction

To assess the interaction of human FOXJ1 and RFX3, the human embryonic kidney cell line 293A was transfected with PGK.EGFP + CMV-Tag2B; PGK.FOXJ1 + CMV-Tag2B; CMV.FLAG-RFX3 + PGK.EGFP or CMV.FLAG-RFX3 + PGK.FOXJ1 expression plasmids using Fugene HD (Roche, Indianapolis, IN). Forty eight hr post-transfection, transfected and untransfected cells were harvested and lysed as described above. Proteins were immunoprecipitated overnight at 4°C with 50 µl of EZview Red Anti-FLAG M2 affinity gel (Sigma, St. Louis, MO). After binding, the beads were collected by centrifugation at 400 x g at 4°C in a Eppendorf 5415C microcentrifuge and washed five times for 5 min each with RIPA

buffer at 4°C. Bound complexes were released from the beads by elution with FLAG peptide (Sigma, St Louis, MO) and subsequently boiled for 10 min in the appropriate volume of 1.5x SDS sample buffer. The released proteins were assessed by SDS-PAGE.

Statistical Analysis

All data in this study are presented as mean ± standard error. Statistical comparisons between continuous variables were calculated using an unpaired, two-tailed t test with unequal variance. A p-value <0.05 was considered to be significant. Average expression values were calculated from normalized expression levels for ALI day 0 cultures. Relative mRNA expression levels and all statistical tests were calculated using MS Excel 2008 (Microsoft).

Additional Data References

1. Ammous,Z, Hackett NR, Butler MW, Raman T, Dolgalev I, O'Connor TP, Harvey BG, Crystal RG: **Variability in small airway epithelial gene expression among normal smokers**. *Chest* 2008, **133:** 1344-1353

2. Hackett,NR, Heguy A, Harvey BG, O'Connor TP, Luettich K, Flieder DB, Kaplan R, Crystal RG: **Variability of antioxidant-related gene expression in the airway epithelium of cigarette smokers**. *Am J Respir Cell Mol Biol* 2003, **29:** 331-343

3. Hackett,NR, Shaykhiev R, Walters MS, Wang R, Zwick RK, Ferris B, Witover B, Salit J, Crystal RG: **The human airway epithelial Basal cell transcriptome**. *PLoS One* 2011, **6:** e18378-

4. Livak,KJ, Schmittgen TD: **Analysis of relative gene expression data using real-time quantitative PCR and the 2(-Delta Delta C(T)) Method**. *Methods* 2001, **25:** 402-408

5. Watanabe,M, Boyer JL, Crystal RG: **AAVrh.10-mediated genetic delivery of bevacizumab to the pleura to provide local anti-VEGF to suppress growth of metastatic lung tumors**. *Gene Ther* 2010, **17:** 1042-1051

6. Follenzi,A, Sabatino G, Lombardo A, Boccaccio C, Naldini L: **Efficient gene delivery and targeted expression to hepatocytes in vivo by improved lentiviral vectors**. *Hum Gene Ther* 2002, **13:** 243-260
