# Supplementary material for: RFX3 Modulation of FOXJ1 regulation of cilia genes in the human airway epithelium
Source: Respir Res. 2013 Jul 3;14(1):70. doi: 10.1186/1465-9921-14-70 (PMC3710277; doi:10.1186/1465-9921-14-70)
Supplement: Additional file 2: Table S1 — Ciliated Cell-associated Genes. [file 1465-9921-14-70-S2.pdf]

Additional File 2: Table S1. Ciliated Cell-associated Genes

Gene symbol	Gene name	Function	References
CETN2	Centrin 2	Ca^{2+}-binding protein, component of centrioles	[1-3]
DNALI1	Dynein, axonemal, light intermediate chain 1	Component of dynein arms	[3-5]
DNAH11	Dynein, axonemal, heavy chain 11	Component of dynein arms	[4]
DNAI1	Dynein, axonemal, intermediate chain 1	Component of dynein arms	[4-6]
EFHC1	EF-hand domain (C-terminal) containing 1	Ca^{2+}-binding protein, abundant in cilia and flagella	[1,3,5]
SPAG6	Sperm associated antigen 6	Component of central pair complex	[3,6,7]
TEKT1	Tektin 1	Component of cilia microtubules	[1,5,8]
TEKT2	Tektin 2	Component of cilia microtubules	[5,6,8]
TUBA1A	Tubulin, alpha 1A	Component of cilia microtubules	[3]

Additional File 2: Table S1 References

1. Yu,X, Ng CP, Habacher H, Roy S: **Foxj1 transcription factors are master regulators of the motile ciliogenic program**. *Nat Genet* 2008, **40:** 1445-1453

2. Laoukili,J, Perret E, Middendorp S, Houcine O, Guennou C, Marano F, Bornens M, Tournier F: **Differential expression and cellular distribution of centrin isoforms during human ciliated cell differentiation in vitro**. *J Cell Sci* 2000, **113 (Pt 8):** 1355-1364

3. Ross,AJ, Dailey LA, Brighton LE, Devlin RB: **Transcriptional profiling of mucociliary differentiation in human airway epithelial cells**. *Am J Respir Cell Mol Biol* 2007, **37:** 169-185

4. Becker-Heck,A, Zohn IE, Okabe N, Pollock A, Lenhart KB, Sullivan-Brown J, McSheene J, Loges NT, Olbrich H, Haeffner K, Fliegauf M, Horvath J, Reinhardt R, Nielsen KG, Marthin JK, Baktai G, Anderson KV, Geisler R, Niswander L, Omran H, Burdine RD: **The coiled-coil domain containing protein CCDC40 is essential for motile cilia function and left-right axis formation**. *Nat Genet* 2011, **43:** 79-84

5. Thomas,J, Morle L, Soulavie F, Laurencon A, Sagnol S, Durand B: **Transcriptional control of genes involved in ciliogenesis: a first step in making cilia**. *Biol Cell* 2010, **102:** 499-513

6. Stubbs,JL, Oishi I, Izpisua Belmonte JC, Kintner C: **The forkhead protein Foxj1 specifies node-like cilia in Xenopus and zebrafish embryos**. *Nat Genet* 2008, **40:** 1454-1460

7. Sapiro,R, Kostetskii I, Olds-Clarke P, Gerton GL, Radice GL, Strauss III JF: **Male infertility, impaired sperm motility, and hydrocephalus in mice deficient in sperm-associated antigen 6**. *Mol Cell Biol* 2002, **22:** 6298-6305

8. Amos,LA: **The tektin family of microtubule-stabilizing proteins**. *Genome Biol* 2008, **9:** 229
