# Supplementary figures and images for: RFX3 Modulation of FOXJ1 regulation of cilia genes in the human airway epithelium
Source: Respir Res. 2013 Jul 3;14(1):70. doi: 10.1186/1465-9921-14-70 (PMC3710277; doi:10.1186/1465-9921-14-70)

A. Ciliated cell

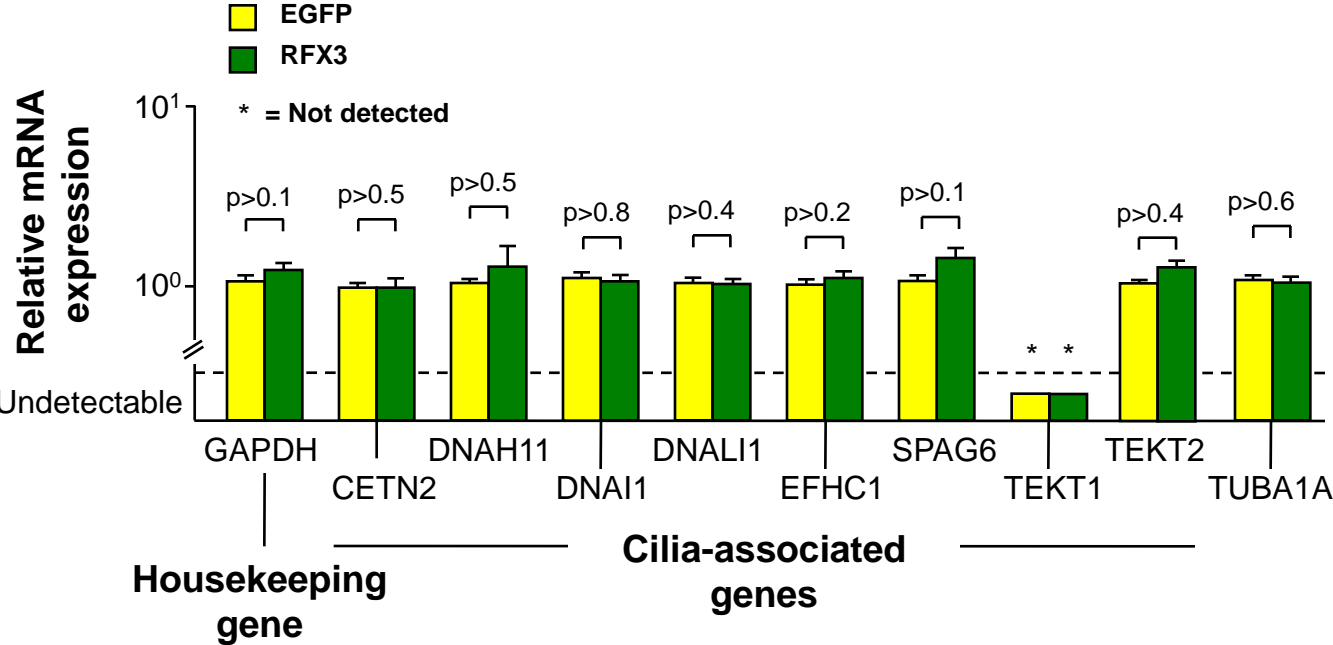

B. Basal cell

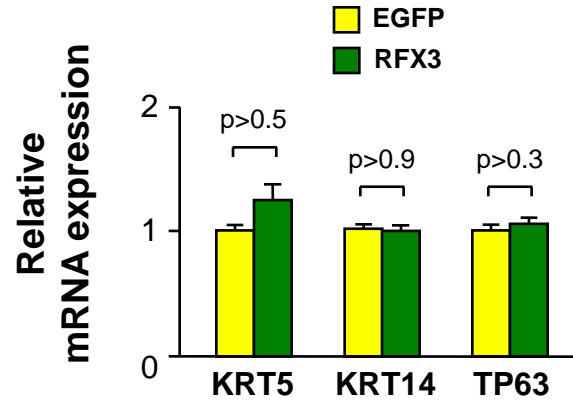

C. Secretory cell

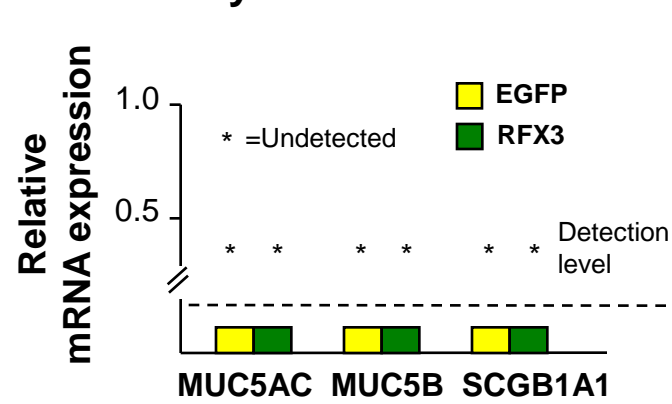

Supplement: Additional file 3: Figure S1 — Expression of cilia-related genes in primary human basal cells transfected with either control EGFP or RFX3 expression plasmids. All data is based on TaqMan quantitative real-time RT-PCR 48 hr after transfection. A. Ciliated cell-associated genes, including CETN2, DNAH11, DNAI1, DNALI1, EFHC1, SPAG6, TEKT1, TEKT2, and TUBA1A. B. Basal cell markers KRT5, KRT14 and TP63. C. Secretory cell markers MUC5AC, MUC5B and SCGB1A1. Bars represent mean ± standard error of pooled data from replicates of a minimum of three individual experiments with cells from different subjects assessed 48 hr after transfection. [file 1465-9921-14-70-S3.pdf]

A. Differentiation

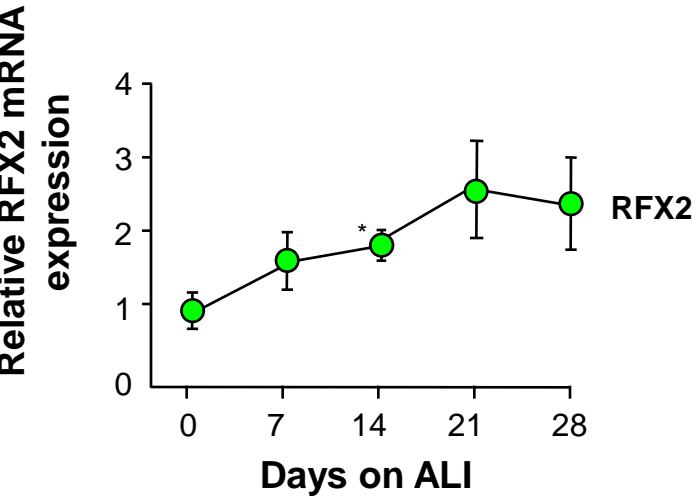

B. mRNA

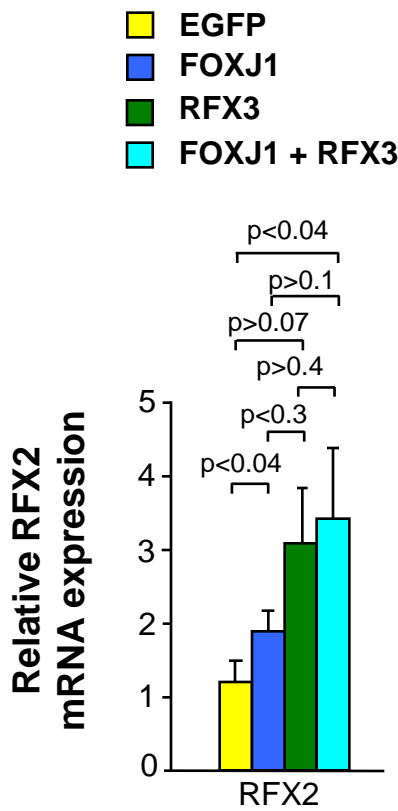

C. Promoter activity

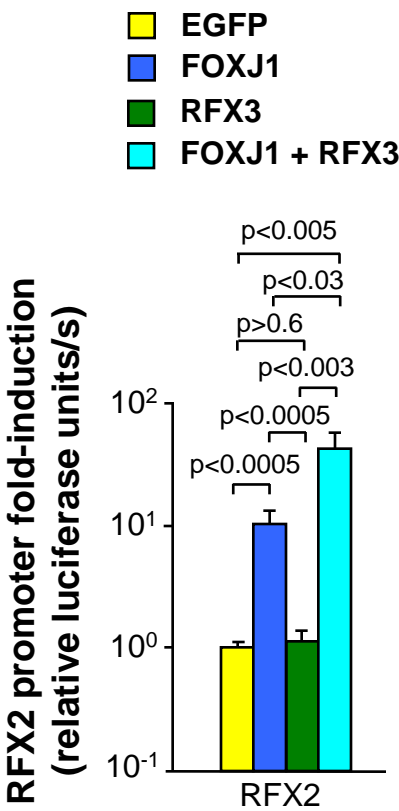

Supplement: Additional file 4: Figure S2 — RFX2 in human basal cell differentiation into ciliated cells. Shown is the temporal expression of RFX2 during basal to ciliated cell differentiation on ALI and the RFX2 mRNA expression and promoter activity in basal cells transfected with FOXJ1 and / or RFX3. A. RFX2 mRNA expression during basal cell differentiation on ALI over 28 days (n=3 per time point). The data was generated by TaqMan quantitative real-time RT-PCR analysis * p<0.05 compared to GAPDH. B. TaqMan quantitative real-time RT-PCR assessment of the relative RFX2 mRNA expression in basal cells transfected with control EGFP, FOXJ1, RFX3 or FOXJ1+ RFX3 expression plasmids. C. Firefly luciferase activity in basal cells transfected with control EGFP, FOXJ1, RFX3 or FOXJ1+ RFX3 expression plasmids together with a firefly luciferase reporter gene plasmid driven by the RFX2 promoter. The data were normalized for transfection efficiency per well by Renilla luciferase activity from co-transfected Renilla luciferase plasmid (pTK-RL). Bars represent mean ± standard error of pooled data from replicates of three individual experiments with cells from different subjects assessed 48 hr after transfection. [file 1465-9921-14-70-S4.pdf]
